# Is There a Space-Based Technology Solution to Problems with Preclinical Drug Toxicity Testing?

**DOI:** 10.1007/s11095-016-1942-0

**Published:** 2016-05-16

**Authors:** Timothy Hammond, Patricia Allen, Holly Birdsall

**Affiliations:** Medicine Service Line/Nephrology Section, Durham VA Medical Center, Building 15, Room 109, 508 Fulton Street, Durham, North Carolina 27705 USA; Nephrology Division, Department of Internal Medicine, Duke University School of Medicine, Durham, North Carolina 27705 USA; Space Policy Institute, Elliott School of International Affairs, Washington, District of Columbia 20052 USA; Office of Research & Development, Department of Veterans Affairs, Washington, District of Columbia 20420 USA; Department of Otorhinolaryngology, Baylor College of Medicine, Houston, Texas 77030 USA; Department of Immunology, Baylor College of Medicine, Houston, Texas 77030 USA; Department of Psychiatry, Baylor College of Medicine, Houston, Texas 77030 USA

**Keywords:** drug metabolism, hepatocyte, space, suspension culture

## Abstract

Even the finest state-of-the art preclinical drug testing, usually in primary hepatocytes, remains an imperfect science. Drugs continue to be withdrawn from the market due to unforeseen toxicity, side effects, and drug interactions. The space program may be able to provide a lifeline. Best known for rockets, space shuttles, astronauts and engineering, the space program has also delivered some serious medical science. Optimized suspension culture in NASA’s specialized suspension culture devices, known as rotating wall vessels, uniquely maintains Phase I and Phase II drug metabolizing pathways in hepatocytes for weeks in cell culture. Previously prohibitively expensive, new materials and 3D printing techniques have the potential to make the NASA rotating wall vessel available inexpensively on an industrial scale. Here we address the tradeoffs inherent in the rotating wall vessel, limitations of alternative approaches for drug metabolism studies, and the market to be addressed. Better pre-clinical drug testing has the potential to significantly reduce the morbidity and mortality of one of the most common problems in modern medicine: adverse events related to pharmaceuticals.

## The Problem with Pre-Clinical Drug Testing

To develop and market a new drug, companies must prove both efficacy and safety. It is clearly more cost effective to identify and disqualify toxic alternatives as early in the development process as possible. *In vitro* models for ADME/Tox (absorption, distribution, metabolism and excretion & toxicology) screening have been the holy grail of drug development (1). Not only are *in vitro* systems more cost effective than *in vivo* testing, but they support the guidelines of the National Research Council and the EPA calling for refinement, reduction and replacement to minimize the use of *in vivo* testing in animals (2).

The liver is the major site of drug metabolism and degradation *in vivo*. 5–10% of adverse drug reactions are the result of liver toxicity and a third of all post-market drug withdrawals are because of liver toxicity (3). The central role of the liver has led to the use of liver cells (hepatocytes) as a major choice for *in vitro* testing systems (1,4). The FDA has already found drug testing with hepatocyte cell culture to be an acceptable preclinical tool (5).

Despite extensive screening, a surprising number of drug failures are still not recognized until late stage clinical trials, after there has been significant investment in the development of the drug candidate (6–9). A recent study found that about 19% of the drugs that failed in Phase II clinical trials and 21% of the drugs that failed in Phase III clinical trials were failures due to safety issues (6,7). One company estimates that clinical failures due to liver toxicity cost them more than $2 billion over the last decade (10). Thus there is a renewed emphasis on earlier and more accurate toxicology evaluation as one way to increase future success and avoid adverse clinical reactions (11)

An ideal *in vitro* hepatocyte model would include cells with prolonged robust biosynthetic capacity (e.g. production of albumin) and normal basal and inducible levels of biotransforming enzymes. Key hepatic biotransforming enzymes include those that metabolize drugs through Phase I (oxidation, reduction and hydrolysis) and/or Phase II (by conjugation of functional groups) processes. An ideal *in vitro* liver model would also recapitulate the organoid structure of the intact organ *in vivo* where hepatocytes cluster to form channels called bile canaliculi into which they secrete their products.

Current *in vitro* liver models fall short of these ideals in many ways. Liver slices lose key metabolic enzymes within hours (2,12). Immortalized hepatocytes remain viable over longer periods of time, but have lower liver specific enzymes than primary cells. Furthermore, cell lines only reflect the phenotype of a single donor and may miss key variants in the human population. Induction of stem cells to provide a continuous supply of hepatocytes is appealing, but efforts to date have been unable to generate hepatocytes with a stable expression of relevant enzymes (2). At present, primary human hepatocytes represent the “gold standard” for preclinical *in vitro* metabolism and toxicity studies (13). Hepatocytes grown as single cell suspensions lose polarity, integrity and differentiation (8,9,14). Primary hepatocytes adherent to plastic dishes are the most commonly used model but these also begin to de-differentiation within 24–48 hours.

## Is There a Space-Based Technology Solution to the Clinical Drug Toxicity Testing Problem?

Three dimensional culture systems are being extensively explored as a means of extending differentiation and function of hepatocytes to better reproduce the microenvironment of the intact liver (14–19). A variety of initiatives have explored the use of automated, microfluidic, and organ-on-a-chip approaches (14–20). Hepatocytes grown in gels or on various bead/fiber scaffoldings display increased and sustained functionality (21–23). However, the inability to adequately oxygenate the cells continues to be a major limiting factor (2). This is where culture techniques, originally designed for space flight studies, may offer a distinct advantage (14,24–30). Hepatocytes grown on Earth in NASA’s rotating wall vessel suspension culture vessels form three-dimensional colonies that maintain their function for at least several weeks (14,15,24). These cell colonies closely resemble and function like natural cells in the human body, which makes them excellent candidates for preclinical drug testing (14,24–30).

The question to be addressed is whether space flight derived technologies have the potential to predict and prevent the morbidity, mortality and staggering cost of incidents where current methods fail to detect toxic pharmaceutical side effects premarket? What are the pharmacological, technical and financial tradeoffs inherent is using NASA suspension culture techniques for preclinical ADME/Tox testing? This review focuses on the core of preclinical drug testing: the ability of hepatocytes cultured in the NASA rotating well vessel, a form of suspension culture optimized to minimize shear, to maintain Phase I and Phase II drug metabolizing enzymes, the technology needed, and how this may address the problems with current testing, Finally we address the opportunities to get involved in space-based research.

Through perseverance and development of new technology, man walked on the moon, the space shuttle flew, and the International Space Station was built. The microgravity environment of spaceflight has provided an opportunity for new approaches to pharmacology. For example, microgravity unloads muscles and bones, allowing BigPharma to fly rodents in space and validate new drugs that reverse the effects of muscle and bone unloading following fracture, rehabilitation, or debility (31). Another opportunity is the facilitation of three dimensional organ culture in both real and emulated microgravity (14,15,17). Experiments conducted by NASA’s Biotechnology Group in the 1980’s on cell behavior in microgravity produced startling results—cells suspended in a Rotating Wall Vessel (RWV) bioreactors formed multicellular organoids (32–35). This revolutionized cell culture technology and ultimately led to the production of a life-sustaining RWV-based artificial liver. NASA suspension culture techniques reverse much of the problematic de-differentiation of cells observed during other forms of tissue culture (35–38). The technology has been applied broadly to diverse cell types including cancer cells, prostate, kidney, micro-organisms, biofilms, and plants, to name a few (36,39,40).

## NASA and Optimization of Suspension Culture

**Fig. 1:**
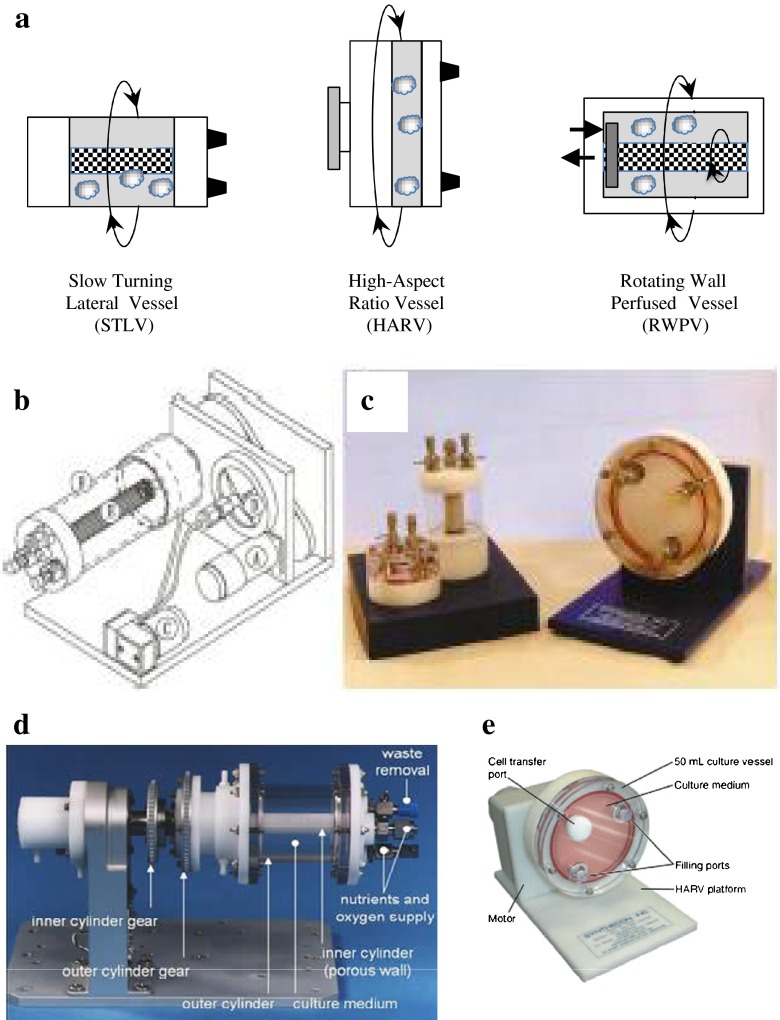
Panel A. A comparison of the Slow Turning Lateral Vessel (STLV), High Aspect Ratio Vessel (HARV) and the Rotating Wall Perfused Vessel (RWPV). Note that the co-axial oxygenator in the STLV is replaced in the HARV by a breathable membrane as the back wall of the vessel. The RWPV can rotate the co-axial oxygenator and outer wall at different speeds, which was needed to mix perfusates in space. Panel B: Schematic of Rotating Wall Vessel. A 24-V direct current motor (**a**) drives a belt that rotates the cylindrical culture vessel (**b**) along its horizontal axis. An air pump (**c**) draws incubator air through a 0.22-micron filter (**d**) and discharges it through a rotating coupling on the shaft that carries the vessel. The oxygenator (**e**) is wrapped around the center post. Reproduced from Ref 35. Panel C: Selected rotating wall vessel volumes and designs. Reproduced from Ref 35. Panel D. The Rotating Wall Vessel adapted for use in space flight. From NASA technical reports. http://www.technovelgy.com/graphics/content/Rotating-Wall-Bioreactor.jpg. Panel E. Schematic of a HARV (from www.Synthecon.com).

Until its reorganization into other programs, NASA’s Biotechnology Group, based at the Johnson Space Center in Houston, TX, modeled the problem of optimizing mechanical culture conditions in suspension culture by minimizing shear and turbulence (33,34,41–46). The rotating wall vessel is essentially a cylinder of fluid that rotates around its long axis. Cells cultured in this fluid will tend to settle under the influence of gravity, but, as the cylinder turns, the cells are lifted back into suspension. By adjusting the rotational speed, a steady state can be achieved where the cells remain in suspension (Fig. 1a). Fluid flow is near solid body or laminar at most operating conditions. This avoids the large shear stresses associated with turbulent flow and allows introduction of controlled and nearly homogenous shear fields. The culture medium is gently mixed by rotation, avoiding the necessity for stirring vanes that damage cells by both local turbulence at their surface and the high flow rates created between the vessel walls and the vanes. Finally, there is no headspace or air gap above the medium. This contrasts with roller bottles that are only partially filled with medium. In roller bottles, air in the headspace creates turbulence and secondary bubble formation in the culture medium, which are both potent sources of extra shear and turbulence.

The reduction of shear and absence of turbulence unique features of the rotating wall vessel and allow cells to aggregate into organoids. Shear in a rotating wall vessel depends directly on the square of the cells’ radius, gravity, and the difference in density between the cells and the culture medium. Shear is inversely related to the viscosity of the culture medium (33,35). Unfortunately density and viscosity of the culture medium are not independent, so there are limits to how much shear stress on cells can be reduced in the vessel by matching culture medium density to the cell density. Hence, by removing the direct effect of gravity, space affords a cell culture milieu very close to zero shear.

The original rotating wall vessel, known as the slow turning lateral vessel (STLV), was shaped like a soup can, and rotated along its long axis (Fig. 1a and b). The components included the vessel itself, a coaxial oxygenator, an air pump, a stand with inbuilt geared rotor, and a tachometer/power supply to control rotation speed. Average rotation speeds for mammalian cells were about 10 revolutions per minute. The coaxial oxygenator and the outer wall may rotate at the same slow (10–16 rpm) rate, avoiding and/or minimizing shear from differential rotation (see below).

Gas exchange in the STLV was limiting for rapidly growing cells. Hence a new design was manufactured in a dinner plate configuration with the entire back becoming a breathable membrane. Known as the high aspect rotating vessel (HARV) (Fig. 1a, c, & e). This design greatly improves gas exchange but still needs an air pump, a stand with inbuilt geared rotor, and a tachometer/power supply to control rotation speed. Both the STLV and HARV have rubber seals that need to be autoclaved at a different temperature to the other cell culture components. The STLV and HARV maintain, and in some cases recover, tissue specific cell differentiation (20,35), but the multiple components with strict engineering tolerances are expensive and labor intensive to load, re-feed, and operate.

When the STLV was adapted for utilization in space, initial flight experiments posed some unanticipated engineering challenges. In space not only is gravity dramatically reduced but convection is reduced as well. The hope was to grow large cell aggregates and induce vascularization. The space flight unit continuously fed the cell cultures via a perfusion loop (Fig. 1d). Studies with colored beads and media showed that the perfused nutrients flowed along the walls with minimal admixture with the gas and nutrient depleted media surrounding the cells. Rotating the vessel induces minimal mixing. The problem was solved by differential rotation of the central coaxial oxygenator and the outer vessel wall. Differential rotation on the ground reintroduces shear (35), but also greatly increases vessel complexity and cost.

The next big challenge was bubbles. Both the STLV and HARV have no headspace free of media, as even a single bubble disrupts laminar flow, and the resulting turbulence damages almost all mammalian cells. The current generation of spaceships purposely have quite low humidity to minimize the dramatic condensation caused by differential heating of the vehicle sides by sun exposure and shadow. In the low humidity, all attempts to seal the STLV flight hardware failed to prevent bubble formation, as water evaporated through perfusion lines and wall materials. The engineering answer to bubble formation was to make the front face of the HARV slightly domed. Bubbles migrated to the peak of the dome and were easily removed intermittently. However, this again induced turbulent rather than laminar flow. Engineering again solved this problem as a small sculpted button on the back face of the HARV, rotating at a different speed to the HARV compensated for the turbulent forces and reintroduced laminar flow.

## The Case for Using RWV-Cultured Hepatocytes for ADME/Tox Testing

- The rotating wall vessel has shown significant promise for improving ADMET testing with hepatocytes. Hepatocytes grown on Earth in NASA’s Rotating Bioreactor form three-dimensional colonies that maintain their function for at least several weeks (14,15,24). The most dramatic proof of this approach was the RWV-based artificial liver that was able to sustain a hepatectomized horse (47) and http://spacesciencesinc.org/pdf/kelleher_testcases.pdf.

Rat hepatocytes have been cultured initially as spheroids on culture plates and then transferred into a HARV for further culturing (14). Morphological evaluation based on electron microscopy showed that hepatocyte spheroids cultured for 30 days in the HARV had a compact structure with tight cell-cell junctions, numerous smooth and rough endoplasmic reticulum, intact mitochondria, and bile canaliculi lined with microvilli. The viability and differentiated properties of the hepatocytes cultured in the HARV were further substantiated by the presence of both phase I oxidation and phase II conjugation drug-metabolizing enzyme activities, as well as albumin synthesis to a greater degree than 2-D controls. Homogenates prepared from freshly isolated hepatocytes and hepatocytes cultured in the HARV showed similar cytochrome P450 2B activities measured as pentoxyresorufin-O-dealkylase and testosterone 16-beta-hydroxylase. Further, intact hepatocytes cultured in the HARV were found to metabolize chlorzoxazone to 6-hydroxychlorzoxazone; dextromethorphan to dextrorphan, 3-methoxymorphinan, and 3-hydroxymorphinan; midazolam to 1-hydroxymidazolam and 4-hydroxymidazolam; and 7-hydroxycoumarin to its glucuronide and sulfate conjugates far better than 2D controls (14).

Several others lines of evidence support the contention that the rotating wall vessel maintains functional hepatocytes. Monolayer and spheroid culture of Hep G2 human liver hepatocellular carcinoma cell line cells demonstrated distinct global gene expression patterns and functional phenotype (16). The molecular mechanisms underlying the enhanced functions of three-dimensional hepatocyte aggregates including a key role for a diverse array of hepatocyte-specific functional genes with significant over-representation of hepatocyte nuclear factor 4α (Hnf4a) binding sites in their promoters. Other rotating wall vessel studies confirm that the hepatocytes in the reconstituted 3D tissue are capable of producing albumin and storing glycogen, forming bile canaliculi between hepatocytes with complicated tubular branches and express elevated levels of mature hepatocyte genetic markers compared to hanging droplet and spinner flask controls (17). But perhaps most importantly, 3D spheroid cultures of HepG2 cells were far superior to 2D cultures in terms of how well their data correlated with *in vivo* lethal blood plasma levels (18). This suggests that 3D hepatic spheroids grown in the rotating wall vessel have excellent potential to predict hepatic toxicity.

## Adapting RWV Technology for ADMET Testing

Although promising in concept as a model for measuring drug metabolism by hepatocytes, current RWV hardware is too expensive and labor intensive to support the high throughput needs of the pharma industry. Current RWVs require expensive hardware and are labor-intensive to load, maintain, and harvest. These factors make current widespread utilization of RWVs cost-prohibitive. Instead, industry has turned to using human hepatocytes in polystyrene plates for drug metabolism studies, with cells on the edge of their useful life due to loss of metabolic enzymes.

However the cost and complexity equation is changing rapidly. The advent of new manufacturing techniques such as 3D printing, in combination with new materials, developed just in the last few years (48–50), have set the stage for a new revolution in cell culture that can be applied to preclinical drug testing. Further, much simpler approaches have been applied to the STLV and HARV cell culture techniques designed to minimize shear on cultured cells. For instance, the vessels can be placed on a device similar to a large hot dog roller, removing the need for individual rotators, tachometers, and power supplies. Costs plummet. To simplify culture procedures, modern self-sealing ports used in clinical medicine replace open Luer locks. New materials provide more hydrophobic membranes for better gas exchange. Inexpensive microfabrication and 3D printing replace labor-intensive use-by-use seal installations and assembly requirements.

## Other Drug Metabolism Studies Show Promise of the ISS

We have flown genetically engineered yeast on three flight missions and found that microgravity provides a cancer-relevant context that extends and complements current models. STS-135 experimental data demonstrated a unique chemogenomic fingerprint of the oxidation status of yeast cells in microgravity for drug pathway analysis, characterized by specific changes in mitochondrial and ribosomal respiratory function with minimal stress response. Microgravity reduces convection and allows reproduction of redox states and gas levels in yeast cells that mimic the microenvironment of tumors (51). Altered redox potential with changes in reactive oxygen species is difficult to model in ground-based cultures because gravity-dependent convection delivers oxygen and purges carbon dioxide from the yeast cells in both solid and liquid culture. Thus microgravity allows yeast-based modeling of drug pathways in ways that are not currently achievable by any other means in ground-based studies. The baker’s yeast *Saccharomyces cerevisiae* has 70% homology with the human genome, and a data bank of >3200 responses to drugs, small molecules, and physiological stresses allows space based data to be tested for unique phenotypic findings (52).

## Opportunities and Barriers

The International Space Station is improving the portfolio of available instrumentation on board over time. You can culture cells in incubators and glove boxes, use centrifuge controls, isolate RNA, perform microscopy and utilize many other technologies (https://en.wikipedia.org/wiki/Scientific_research_on_the_International_Space_Station). Diverse flight hardware is available from NASA as well as US commercial groups such as Bioserve Space Technologies (http://www.colorado.edu/engineering/BioServe/), Techshot (Techshot.com), and NanoRacks (Nanoracks.com), as well as several international partners (http://global.jaxa.jp; http://m.esa.int/ESA; http://www.energia.ru/english/; http://www.asc-csa.gc.ca/eng/) The barriers to involvement include funding, a tolerance for regulatory paperwork, and a robust scientific plan.

## Getting involved

Come to the party. One thing you learn reviewing grants is that there are amazingly creative and insightful scientists in the community. We invite and encourage you to apply space resources, and improve space-based research. There are many funding resources available including, but not limited to:

- Working directly with a commercial partner with a specific problem in partnership with the NASA ISS – Commercial Space Utilization Office found at http://www.nasa.gov/mission_pages/station/research/nlab/index.html, the Center for the Advancement of Science in Space (CASIS) at http://www.iss-casis.org/Opportunities/Solicitations.aspx, or the NASA Small Business Innovation Research (SBIR/ Small Business Technology Transfer programs (STTR) found at http://sbir.nasa.gov/solicitations.
- NASA has multiple branches supporting biomedical research all of which are announced in the NASA Solicitation and Proposal Integrated Review and Proposal Evaluation System (NSPIRES), which can be found at https://nspires.nasaprs.com/external/.
- The Canadian Space Agency has a variety of announcements of opportunities to be found at http://www.asc-csa.gc.ca/eng/ao/.
- The National Institutes of Health (NIH) and NASA have sponsored joint research initiatives such as http://www.niams.nih.gov/News_and_Events/NIH_NASA_Activities/
- The Japan Aerospace Exploration Agency (JAXA) announces opportunities at multiple sites such as http://www.isas.jaxa.jp/e/enterp/sbms/index.shtml
- The European Space Agency (ESA) provides research opportunities in collaboration with a ESA scientist as announced at http://www.esa.int/Our_Activities/Human_Spaceflight/Research/Opportunities_for_research_with_International_Space_Station

## Recommendations

Cells respond to perturbations. The question is not whether cells change in space, but rather what is unique about the space-based environment, and how can that knowledge be utilized in ground-based applications? Ground-based controls are critical and databases of responses from space-based studies can be used to put uniqueness and utility into context]. In the United States there is a Presidential Executive Order to make all data collected with Federal funding publically available (https://www.whitehouse.gov/the-press-office/2013/05/09/executive-order-making-open-and-machine-readable-new-default-government-). As almost all US space data includes some element of NASA funding, all data is/will be publically available on US Government websites, in addition to what can be retrieved from journal or investigator websites. We recommend you download, reanalyze, and utilize this data freely.

The International Space Station has a life limited by engineering principles on the materials, and the political and financial will of the participants. We should not hesitate in our drive to investigate if the near zero shear of space-based hepatic cultures allows better prediction of drug interactions and toxicity than current models, to improve safety profile of preclinical drug testing, and address the current limitations on ADME/Tox testing, which can result in massive morbidity and mortality.
